# Psychometric evaluation of the Arabic language version of the Birchwood Insight Scale in patients with schizophrenia

**DOI:** 10.1186/s12888-024-05657-8

**Published:** 2024-03-27

**Authors:** Feten Fekih-Romdhane, Zeinab Bitar, Fadila Al Mouzakzak, Ghinwa Abilmona, Oussama Dahdouh, Souheil Hallit

**Affiliations:** 1 Faculty of Medicine of Tunis, Tunis El Manar University, Tunis, Tunisia; 2 The Tunisian Center of Early Intervention in Psychosis, Department of psychiatry “Ibn Omrane”, Razi hospital, Manouba, 2010 Tunisia; 3 Univ Rennes, Inserm, EHESP, Irset (Institut de recherche en santé, environnement et travail), Rennes, UMR_S 1085, F-35000 France; 4 Faculty of Science, Lebanese University, Fanar, Lebanon; 5 Department of Psychiatry, Psychiatric Hospital of the Cross, P.O. Box 60096, Jal Eddib, Lebanon; 6 School of Medicine and Medical Sciences, Holy Spirit University of Kaslik, P.O. Box 446, Jounieh, Lebanon; 7 Applied Science Research Center, Applied Science Private University, Amman, Jordan

**Keywords:** Schizophrenia, Clinical insight, Birchwood Insight Scale, BIS, Arabic, Psychometric properties

## Abstract

**Background:**

Clinical insight (i.e., impaired insight into illness) is increasingly recognized by the scientific community as a significant contributor to an array of psychological and clinical outcomes in schizophrenia. Therefore, its assessment using a reliable, rapid, easy and economic tool is important for clinical practice. This study proposes to investigate the psychometric properties of an Arabic translation of the Birchwood Insight Scale (BIS) in Arabic-speaking chronic patients with schizophrenia. Our objectives were to identify the most adequate factor structure of the BIS among the several measurement models previously proposed in the literature, verify the reliability and measurement invariance of the BIS across sex groups, and explore the concurrent validity of the BIS through examining its patterns of correlations with psychotic symptoms.

**Method:**

One hundred seventeen Arabic-speaking chronic, remitted patients with schizophrenia took part in this study. An Arabic translated version of the BIS and the Positive and Negative Syndrome Scale (PANSS) were administered to participants.

**Results:**

Confirmatory factor analyses (CFA) showed that, after omitting two items with low loadings (items 1 and 2), the unidimensional factor model of the BIS showed good fit indices and a reliability of α = 0.68 and ω = 0.68. However, analyses failed to show good fit for the full-length one-, two-, and three-factor models of the BIS in its Arabic version. Measurement invariance of the Arabic 6-item one-factor BIS was established between males and females at the configural, metric and scalar levels; no statistically significant difference between males and females was found in terms of BIS scores. Finally, BIS scores correlated significantly with the PANSS scores in our sample, thus demonstrating adequate concurrent validity.

**Conclusion:**

This study offers valuable additional psychometric information about the BIS based on results of CFA and other analyses in schizophrenia from a non-Western cultural environment. We believe that making the BIS available in Arabic might benefit clinicians working with Arabic-speaking patients with schizophrenia, open new avenues of research and gain a better knowledge into the nature of clinical insight and its relevance to psychopathology.

**Supplementary Information:**

The online version contains supplementary material available at 10.1186/s12888-024-05657-8.

## Introduction

Insight is conceived as a biopsychosocial multidimensional construct that comprises aspects such as awareness (i.e., the recognition that one has a mental illness), attribution (i.e., the ability to recognize and re-label symptoms as pathological), and action (i.e., the recognition and acceptance of the necessity of treatment) [1, 2]. In line with this multidimensional conceptualization, recent findings from functional neuroimaging studies observed that insight dimensions are differentially mediated by specific brain regions [3]. Insight exists on a continuum, with severity varying from person to person and at different time points over the course of the disease in the same person [4, 5]. It is also assumed to differ across signs and symptoms of the disease [4, 6]. Impaired insight into illness, also referred to as clinical insight and which is the main focus of the present paper, represents a prominent clinical characteristic across various severe mental health conditions, in particular schizophrenia. Previous studies reported estimated prevalence rates of poor insight in patients diagnosed with schizophrenia ranging from 30 to 80%, and is therefore considered a key feature of the disease [7–9].

Impaired insight has consistently been recognized as a major clinical issue in schizophrenia for many reasons. It has, for example, been identified as a strong predictor of antipsychotic medication noncompliance in this population [10, 11]. In addition, evidence from meta-analyses indicated that impaired insight is linked to deficits in neurocognitive and social cognition abilities [12], more severe disorganization and negative symptoms [12], lower quality of life [13], impaired social functioning [14] and psychosocial adjustment [15, 16], unfavorable psychotherapy outcome [17], as well as more negative long-term illness consequences [7]. Although for some researchers insight is regarded as a stable feature [18], a systematic review and meta-analysis of randomized controlled trials found that metacognitive interventions, such as Metacognitive Training and Metacognitive Reflection and Insight Therapy, are effective in improving insight among patients with schizophrenia spectrum disorders [19]. Given the negative effects of limited insight on the disease’s outcomes, and the potential value of metacognitive interventions for improving insight in schizophrenia, feasible methods for clinical practice that could be used in the context of schizophrenia to measure insight are highly needed. Furthermore, measuring insight has potential implications for applying the currently recommended patient-centered approach of recovery and actively involving patients in their healthcare decision-making, as it was found to affect treatment decisional capacity in patients with psychosis [20].

Several scales have been designed and validated with rigorous methods in order to accurately measure the clinical insight construct. Included among these measures are the 74-item Scale to Assess Unawareness of Mental Disorder [21], the Schedule of Assessment of Insight-E [22], and the Schedule of Assessment of Insight, which are semi-structured or structured interviews. Many previous studies opted for the use of a single item, i.e. the G12 item of the semi-structured Positive and Negative Syndrome Scale [23], an approach that might insufficiently reflect the clinical insight construct, its specific aspects and their clinical relevance [24]. Even though all these measures demonstrated satisfactory psychometric characteristics, their length and/or administration method may be an obstacle for data collection in large-scale or multiple time-points studies that operate under severe time and cost constraints. Self-report measures represent a good alternative to obtain the same information in less time, with low cost and burden, while maintaining sound psychometric properties and providing a better control for confounders inherent in respondent-interviewer interaction (such as the tendency to rate individuals with poorer communication skills or lower intelligence as having impaired insight) [25, 26].

One widely used self-report measure of clinical insight in psychotic disorders is the Birchwood Insight Scale (BIS; [27]). The BIS consists of eight items which are rated on a five-point Likert scale ranging from 0 (disagree very much) to 4 (agree very much). The BIS has considerable advantages over other scales used in this population (such as the VAGUS; sample items: “My mental illness has caused me to hear voices that other people cannot hear”, or “I definitely NEED treatment with an antipsychotic medication” [28]), as it is based on a symptom-unspecific wording of items, thus enabling to assess insight transdiagnostically across various mental disorders. In the first validation study, developers initially conceptualized the BIS as a multidimensional instrument aimed at measuring the three insight dimensions advocated by David [2] (awareness of illness, need for treatment and attribution of symptoms). However, they found that all eight items loaded into a unique higher-order dimension accounting for 60% of the total variance and conceived as a measure of overall general insight [27]. In a subsequent study by Trauer and Sacks [29], a three-factor solution was supported in a sample of patients with psychotic disorders, predominantly schizophrenia. Afterwards, a study by Cleary et al. [30] showed that the unidimensional model of the BIS has best fit to the data after removing one item. Yet more recently, other psychometric studies suggested that the two components awareness of illness and awareness of the need for treatment produce a single factor of insight [1, 31]. In sum, the construct validity of the BIS yielded mixed findings, and only limited research exists to date investigating its psychometric qualities despite its large use worldwide.

### Rationale of the present study

As clinical insight is increasingly recognized by the scientific community as a significant contributor to an array of psychological and clinical outcomes in schizophrenia, its assessment using a reliable, rapid, easy and economic tool like the BIS is important for clinical practice. Besides, failing to accurately measure impaired insight poses a major barrier to elucidating its underlying etiology in schizophrenia and to the development of effective interventions [32]. Surprisingly, however, there is yet no or only very limited information, in particular from non-Western countries, on psychometric properties of the BIS. Previous validation studies involved patients with psychotic disorders from UK (which revealed strong face validity, three-factor structure, Cronbach α of 0.75, good interrater reliability and concurrent validity) [27], US (which revealed adequate construct validity, a one-factor structure, and Cronbach α of > 0.70) [30], Norway (three-factor structure, good construct validity and convergent validity, Cronbach α of > 0.70) [33, 34], and lately, Korea (which showed 2 factors, good internal consistency test-retest reliability and Concurrent validity) [31]. However, there is still no Arabic version of the BIS in the scientific literature with evidence of validity and reliability. It is of note that an Arabic translation of the scale has previously been used in research without validation [35, 36]. To bridge this gap, and contribute the literature in this field, this study proposes to investigate the psychometric properties of an Arabic translation of the BIS in Arabic-speaking chronic patients with schizophrenia. Our objectives were the following: (1) to identify the most adequate factor structure of the BIS among the several measurement models previously proposed in the literature (one, two or three factors), (2) to verify the reliability and measurement invariance of the BIS across sex groups, and (3) to explore the concurrent validity of the BIS through examining its patterns of correlations with psychotic symptoms.

## Methods

### Sample and procedure

This cross-sectional study has been conducted during August and October 2023. The target sample was set as inpatients of the Psychiatric Hospital of the Cross, Jal Eddib (suburbs of the capital Beirut), Lebanon, with the following inclusion criteria: (1) age of 18 years and over, (2) with a schizophrenia or a schizoaffective disorder diagnosis following the Diagnostic and Statistical Manual of Mental Disorders (DSM-5) criteria [35]; (3) at chronic stage of the disease, defined as with more than 1 year of illness duration [37]; and institutionalized in the above-mentioned long-stay hospital for more than one year (We considered stable chronic patients those who were hospitalized for a duration of one year or more and whose medications did not change over a three-month period; the detailed description of the study population can be found elsewhere [35, 38]); (4) experiencing either partial or total recovery, this choice has been made as personal recovery represents a longitudinal process occurring in stages [39, 40]; and (5) able to give their free and informed consent to participate after study objectives and general instructions were thoroughly explained to them (in case of inability to consent a family member did). Patients who do not fulfil the inclusion criteria and those who refused to participate were excluded from the study.

#### Minimal sample size calculation

A sample between 24 and 160 participants was needed for the confirmatory factor analysis based on a previous study that suggested a minimum sample ranging from 3 to 20 times the number of the scale’s variables [41].

### Measures

#### Demographic and clinical characteristics

Data were gathered during a face-to-face interview of around 30–45 min with all participants. The questionnaire consisted of a first section containing information on socio-demographics, including age, sex (male/female), education level (Primary/secondary/university), marital status (single/married/separated/divorced/widowed). The duration of illness and duration of hospitalization were obtained from medical records of each patient. In addition, two measures were administered to all participants.

#### The Birchwood Insight Scale (BIS)

The BIS is a self-report scale composed of eight items scored from 0 to 4. Total scores range from 0 to 32, with greater scores reflecting more impaired insight [27]. The scale underwent translation into Arabic using a rigorous forward-backward method following international guidelines [42]. An independent Lebanese translator, unrelated to the study, initially translated the English content into Arabic. The Arabic version was subsequently translated back into English by a proficient Lebanese psychologist with full proficiency in English. Any literal or context-specific translations were reconciled by the translation team. To ensure translation accuracy, a panel of experts, consisting of the research team, a psychologist, a psychiatrist, and the two translators, scrutinized both the original English versions and the translated versions, rectifying any disparities. A pilot study was done on 30 participants to make sure all questions are clear. Since the questions were well understood by patients in the pilot study, those results were included in the final database and were not analyzed separately.

#### The Positive and Negative Syndrome Scale (PANSS)

The PANSS is an interviewer-rated measure that was used to assess clinical symptoms of psychosis [23]. This is a 30-item measure divided into three dimensions: positive symptoms (7 items), negative symptoms (7 items), and general psychopathology (16 items) [23]. Items are rated on a scale ranging from 1 (absence of symptoms) to 7 (extremely severe symptoms). Higher scores reflect more severe symptoms. The Arabic validated version of the PANSS was used [43]. The present sample yielded Cronbach’s alpha values for total PANSS scores of 0.86.

### Data analysis

The CFA was performed using RStudio (Version 1.4.1103 for Macintosh) (R, [44]) and the Lavaan [45] and semTools [46] packages. We used the weighted least squares means and variance adjusted (WLSMV) estimation method, which is more appropriate for ordinal data. To check if the model was adequate, several fit indices were calculated: the normed model chi-square (χ²/df), the Steiger-Lind root mean square error of approximation (RMSEA), the standardized root mean square residual (SRMR), the Tucker-Lewis Index (TLI) and the comparative fit index (CFI). Values ≤ 5 for χ²/df, ≤ 0.08 for RMSEA, ≤ 0.05 for SRMR, and 0.90 for CFI and TLI indicate good fit of the model to the data [47]. There was no multicollinearity between the variables entered in the model since the VIF values were < 2.5.

### Sex invariance

To examine sex invariance of the BIS scores, we conducted multi-group CFA [48] using the total sample. Measurement invariance was assessed at the configural, metric, and scalar levels [49]. Configural invariance implies that the latent scales variable(s) and the pattern of loadings of the latent variable(s) on indicators are similar across gender (i.e., the unconstrained latent model should fit the data well in both groups). Metric invariance implies that the magnitude of the loadings is similar across gender; this is tested by comparing two nested models consisting of a baseline model and an invariance model. Scalar invariance implies that both the item loadings and item intercepts are similar across gender and is examined using the same nested-model comparison strategy as with metric invariance [50]. We accepted ΔCFI ≤ 0.010 and ΔRMSEA ≤ 0.015 or ΔSRMR ≤ 0.010 as evidence of invariance [48].

### Further analysis

We used Cronbach’s α coefficient and McDonald’s ω and Cronbach’s α coefficients to examine reliability, with values greater than 0.70 reflecting adequate composite reliability. Missing values were replaced by the mean of the item. The BIS scores were considered normally distributed according to their skewness and kurtosis values varying between ± 1 [51]. Consequently, the Student t test was used to compare two means. Pearson test was used to correlate those scores with other scores.

## Results

One hundred forty eight patients filled the survey, with a mean age of 57.15 ± 10.77 years and 66.2% males. Other characteristics of the sample can be found in Table 1. The correlation between items is added as Supplementary Table 1.

**Table 1:** Sociodemographic and other characteristics of the patients (*n* = 148)

Sex	
Males	98 (66.2%)
Females	50 (33.8%)
Education level	
Primary	38 (26.7%)
Complementary	44 (31.0%)
Secondary	43 (30.3%)
University	17 (12.0%)
Marital status	
Single	129 (87.2%)
Married	5 (3.4%)
Divorced	12 (8.1%)
Widowed	2 (1.4%)
Age (years)	57.15 ± 10.77
Duration of illness (years) median	30.00
Interquartile range	
25th	19.75
50th	30.00
75th	37.00
Duration of hospitalization (years) median	9.00
Interquartile range	
25th	5.00
50th	9.00
75th	17.00
PANSS total score	61.58 ± 21.91
BIS score- 8 items	6.67 ± 3.66
BIS score- 6 items	5.91 ± 3.35

### Confirmatory Factor Analysis (CFA) of the BIS scale

The fit indices of the one-factor structure were modest as follows: χ^2^/df = 40.24/20 = 2.12, RMSEA = 0.08 (90% CI 0.04, 0.12), SRMR = 0.06, CFI = 0.84, TLI = 0.78. When adding a correlation between items 3 and 5 because of high modification indices, the fit indices became excellent as follows: χ^2^/df = 20.23/19 = 1.06, RMSEA = 0.02 (90% CI 0.001, 0.08), SRMR = 0.04, CFI = 0.99, TLI = 0.99. The loading factors of items 1 and 2 were low (Table 2); therefore, we removed them and re-did the analysis of the one-factor structure. The fit indices were as follows: χ^2^/df = 10.19/8 = 1.27, RMSEA = 0.04 (90% CI 0.001, 0.11), SRMR = 0.04, CFI = 0.98, TLI = 0.97. The reliability was adequate as shown via the alpha (= 0.69) and the omega (= 0.69) coefficients.

The fit indices of the two-factor structure were as follows: χ^2^/df = 32.20/13 = 2.47, RMSEA = 0.10 (90% CI 0.006, 0.14), SRMR = 0.06, CFI = 0.85, TLI = 0.75. The fit indices of the three-factor structure were modest as follows: χ^2^/df = 77.85/23.00 = 3.38, RMSEA = 0.07 (90% CI 0.05, 0.08), SRMR = 0.05, CFI = 0.93, TLI = 0.89.

**Table 2:** Standardised Estimates of Factor Loadings from the Confirmatory Factor Analysis of the Birchwood Insight Scale

Item number	Label	1 Factor	1 Factor without items 1 and 2	2 factor structure		3 factor structure		
				F1	F2	F1	F2	F3
BIS 1	Some of the symptoms were made by my mind.	0.14	-	-	0.13	-	-	0.11
BIS 2	I am mentally well.	0.09	-	0.13	-	0.15	-	-
BIS 3	I do not need medication.	0.56	0.57	0.99	-	-	0.63	-
BIS 4	My stay in hospital was necessary.	0.52	0.51	0.56	-	-	-	0.33
BIS 5	The doctor is right in prescribing mediation for me.	0.60	0.61	0.49	-	-	0.78	-
BIS 6	I do not need to be seen by a doctor or psychiatrist.	0.39	0.39	0.36	-	0.98	-	-
BIS 7	If someone said I had a nervous or mental illness then they would be right.	0.55	0.55	0.51	-	0.30	-	-
BIS 8	None of the unusual things I experienced are due to an illness.	0.53	0.52	-	0.61	-	-	0.86
χ^2^/df		2.12	1.27	2.47		3.38		
RMSEA		0.08	0.04	0.10		0.07		
SRMR		0.06	0.04	0.06		0.05		
CFI		0.84	0.98	0.85		0.93		
TLI		0.78	0.97	0.75		0.89		

χ²/df = normed model chi-square; RMSEA = Steiger-Lind root mean square error of approximation; TLI = Tucker-Lewis Index; CFI = comparative fit index; SRMR = standardized root mean square residual. Values ≤ 5 for χ²/df, ≤ 0.08 for RMSEA, ≤ 0.05 for SRMR, and 0.90 for CFI and TLI indicate good fit of the model to the data

### Sex Invariance of the BIS

We were able to show the invariance across sex of the one-factor model (6 items and 8 items) at the configural, metric, and scalar levels (Table 3). No statistically significant difference between males and females was found in terms of BIS scores (M = 5.91, SD = 3.36 vs. M = 5.90, SD = 3.38, *t*(146) = 0.01, *p* = .989).

**Table 3:** *Measurement Invariance of the Birchwood Insight Scale across sex in the total sample (using one factor model without items one and two)*

Model	CFI	RMSEA	SRMR	Model Comparison	ΔCFI	ΔRMSEA	ΔSRMR
Configural	0.788	0.120	0.087	Configural vs. metric			
Metric	0.781	0.135	0.086		0.007	− 0.015	0.001
Scalar	0.788	0.120	0.087	Metric vs. scalar	0.007	− 0.015	0.001

Note. CFI = Comparative fit index; RMSEA = Steiger-Lind root mean square error of approximation; SRMR = Standardised root mean square residual

### Concurrent validity

The 6-item BIS total score was negatively and weakly correlated with the total PANSS scores (*r* = − .18; *p* = .034).

## Discussion

The BIS is a brief and easy to administer self-report measure, and is growingly used in clinical practice and psychiatric research. The present study proposes to add to the insight literature by investigating the construct validity of an Arabic translation of the BIS using CFA, its concurrent validity using bivariate correlational analyses, internal consistency and cross-sex measurement invariance. In particular, we sought to determine whether earlier findings of BIS’s factor structure can be replicated in a sample of Arabic-speaking patients with schizophrenia from a Middle Eastern country, by testing three different solutions. Findings showed that, after omitting two items with low loadings, the unidimensional factor model of the BIS showed good fit indices and a reliability of α = 0.69 and ω = 0.69. The Arabic 6-item BIS was invariant between males and females, and demonstrated good concurrent validity through its significant negative correlations with PANSS scores.

CFA failed to show good fit for one-, two-, or three-factor models of the BIS in its Arabic version. The single-factor model fit the data significantly better than other tested models after omitting two items from the scale (items 1 and 2). Consistently, a systematic review indicated that factor analyses of measures developed to assess insight as a multidimensional construct have often yielded a unidimensional factor solution [7]. Cleary et al. [30] examined the factor structure of the BIS in two different samples of English-speaking patients with first-episode psychosis and chronic serious mental illnesses; they showed that a one-factor solution was the best-fitting model after eliminating item 1 (“Some of your symptoms are made by your mind”). According to the authors, a possible reason for the poor performance of item 1 (“symptoms made by their mind”) is that it can be interpreted in two different ways by patients, either as reflecting good insight (symptoms require treatment), or as indicating impaired insight (symptoms are not the manifestation of the disease). Subsequently, Cleary et al. [30] called researchers to explore factor structure and “potentially consider eliminating item 1” when using the BIS [30]. Within the particular Arab context, experiences such as “seeing” visions, “hearing” voices, and holding beliefs in spirits (Djinn), possession, and black magic (“evil eye”) might be are less likely to be reported as mental health problems or as “made by own mind” [52, 53]. As for item 2, remitted patients included in this study might perceive themselves as recovered and “mentally well” at the time of scale administration, which does not necessarily reflect a lack of awareness of their illness or limited insight. Besides, being “mentally well” can be interpreted in different ways across cultures, like for example as being in good spiritual/religious health and relationship with God, or as being healthy emotionally and physically [54]. Physical health is culturally valued in certain cultures, where being physically tired can be seen as being mentally unhealthy [55]; whereas religious health has much more value in other contexts (such as Arab countries), where religious activity and involvement is closely tied to feeling mentally well. altogether, we do not think that the omission of items 1 and 2 of the Arabic version of the BIS could potentially reduce both sensitivity and specificity of the scale as our results showed that these items do not really contribute to the overall measurement of insight in patients with schizophrenia.

Broadly in line with our results, Jan et al. [31] used explorative factor analysis and found that the Korean version of the BIS yielded two factors, with Awareness of illness and Need for treatment producing a single factor of insight. Likewise, a recent study aiming at identifying insight dimensions derived from multiple self- and interviewer-rated scales, including the BIS, in a large cohort of patients with schizophrenia demonstrated that the construct of insight is multidimensional in nature, with awareness of illness and awareness of the need for treatment generating a unique factor [1]. In contrast, and as indicated by Lincoln et al. [7], factor analyses of multiple other measures of insight (e.g., [56–58]) found that items pertaining to these two factors are not merged onto a unique dimension. In addition, and using the Norwegian version of the BIS, Jónsdóttir et al. [33] and Büchmann et al. [34] were able to replicate the original three-factor structure in patients with schizophrenia spectrum disorder. It is of note that our findings might have been affected by the sample chosen for the validation, as chronic remitted inpatients in a long-stay psychiatric hospital may perceive and experience insight differently than outpatients or acute-phase patients. They can also be exposed to long-term compulsory treatment and psychoeducation sessions as part of routine care, and consequently acquire a medical vocabulary that may impact the way they respond to items about the disease [59]. As such, and given the scarcity of psychometric data on the BIS, our study raises the need to re-evaluate the factor structure of the Arabic BIS as well as linguistic versions, other than those currently available (such as Chinese or Spanish), using all eight items in larger samples of patients in different settings and at different stages of the disease (e.g., first episode psychosis).

Alpha and omega reliability coefficients were slightly below the generally accepted threshold of 0.70, indicating moderate reliability. This might indicate more measurement error in the scores obtained, potentially leading to less accurate assessments of insight; therefore, researchers and clinicians should interpret BIS scores with caution. In clinical practice, clinicians may need to supplement insight assessments with clinical interviews to obtain a more comprehensive understanding of patients’ insight into their illness. The borderline reliability of the BIS could be influenced by the translation process and cultural adaptation.

Furthermore, measurement invariance of the Arabic 6-item one-factor BIS was established across sex groups at the configural, metric and scalar levels, which implies that the construct validity of the scale is the same across male and female patients, and that group comparisons of self-reported insight scores are indicative of genuine between-sex differences, not contaminated by measurement discrepancies or group specific biases [60, 61]. To our knowledge, this psychometric property of the BIS has not been previously examined for patients with schizophrenia. This is surprising, as there are interesting and relevant research pointing to inconsistent findings on sex differences in the deficit of clinical insight in patients with psychotic disorders, with females presenting with either better [62, 63], worse [64], or comparable [65] levels of insight. In the present sample, no statistically significant difference was found between sex groups in terms of BIS scores, which was in line with findings from several previous studies [66–69]. To clarify these controversial results, testing measurement invariance of the BIS is essential for future research to accurately reflect the insight construct from patients’ perception of insight. Using a measure of insight that exhibits measurement invariance across sex allows to ensure that it is interpreted and used consistently by males and females, and might help implement a more individualized, sex-tailored approach in therapeutic services for schizophrenia.

Finally, BIS scores correlated significantly with the PANSS scores in our sample, suggesting that psychotic symptoms can negatively influence insight of schizophrenia. These findings corroborate those from prior psychometric research of other linguistic versions of the BIS, such as the Norwegian [34] and the Korean [31] versions, and suggest that the Arabic BIS is a valid tool for measuring clinical insight in Arabic-speaking patients with schizophrenia. There is strong evidence that a better insight is associated with less severe positive, negative and general psychopathology symptoms [8]. Clinicians can use this information to better understand how insight can be influenced by symptom presentation in patients, i.e. treatments that alleviate psychotic symptoms could also improve clinical insight levels. Clinicians can use the BIS to routinely assess the poor-insight patient groups, and subsequently offer a range of interventions serving to improve understanding and knowledge of the illness, and the capacity to communicate more clearly and efficiently about the illness. Such interventions need to be monitored for their effectiveness in influencing the levels of insight, using the BIS.

### Study limitations

This study has some limitations that need to be recognized and addressed. Only remitted inpatients with schizophrenia were involved in our study, which may limit the generalization of findings to other patients’ populations, such as those in acute phases of the disease, those in early stages, and those suffering from diseases other than schizophrenia. Future studies still need to validate the Arabic BIS in these groups. Patients had a relatively high mean age of the patients (57.15 years), and mean age has been found to have a strong association with a cognitive decline among patients with schizophrenia [70]. This might have impacted the quality of the answers. Patients were recruited from one hospital in Lebanon, predisposing us to a selection bias. The correlation between BIS and PANSS scores was weak, which might suggest poor concurrent validity. In addition, as only Lebanese patients have been involved, more studies are warranted to explore whether the Arabic BIS can be applied to Arabic-speaking patients from other Arab countries of different social and cultural backgrounds (such as Gulf or North African Arab countries). In addition, the Arabic BIS had borderline reliability and some important psychometric properties were not investigated in this study, such as convergent validity, inter-rater and test-retest reliability. Future research directions should aim at enhancing the scale’s reliability, exploring its sensitivity to change over time, and assessing its predictive validity for treatment outcomes in schizophrenia.

### Implications and future perspectives

This study validates the Arabic-language version of the BIS, which can be used as a self-reported insight measure for Arabic-speaking patients with schizophrenia. Although interviewer-administered measures are valuable in evaluating expressed behaviors and attitudes, self-report scales have the potential to provide clinicians and researchers with important information on perceived internal experiences and opinions of patients with schizophrenia to avoid possible inter-rater biases or raters’ misinterpretation of deficits in cognition/communication as limited insight [26]. The Arabic BIS was demonstrated to be valid, reliable, and suitable for use in clinical practice and research for the assessment of the clinical insight construct. Based on our findings and previous observations, it is suggested that the Arabic version of the BIS should be regarded as a unidimensional scale including six items that load onto a single factor and cover all three dimensions initially proposed by the developers, namely relabeling of symptoms (item 8), awareness of illness (item 7), and need for treatment (items 3, 4, 5, and 6). However, the dimensional properties of the Arabic BIS still need to be investigated in further studies involving patients with different clinical characteristics to confirm our results. In this regard, we support the suggestions of some authors [30] to include more culturally appropriate items to the BIS, while removing the first two items, in order to accurately assess a more multidimensional construct of insight. Lastly, we believe that making the BIS available in Arabic might allow novel evidence-informed techniques and psychotherapeutic interventions serving to enhance insight to be tested in Arab contexts. Providing the Arabic BIS to clinicians and researchers working in Arabic-speaking settings has also the capacity to open new avenues of research and gain a better knowledge into the nature of clinical insight and its relevance to psychopathology.

## Conclusion

The current data provides, for the first time, useful information on the factor structure of the BIS from a sample of chronic patients with schizophrenia in an Arabic-speaking setting. This is helpful information for clinicians and researchers who are ethically compelled to have a certain degree of confidence in a measure and know that it could fulfil their evaluation needs before applying it to patients. The analysis revealed that the one-factor model showed good and acceptable fit with the data set with only six items. It is highly suggested that future psychometric research on the Arabic BIS should consider including additional items to better reflect multidimensionality, as well as other aspects of reliability and validity not undertaken in the current study.

### Electronic supplementary material

Below is the link to the electronic supplementary material.

Supplementary Material 1

## Data Availability

All data generated or analyzed during this study are not publicly available. The dataset supporting the conclusions is available upon request to the corresponding author.
