# Supplementary material for: Psychometric evaluation of the Arabic language version of the Birchwood Insight Scale in patients with schizophrenia
Source: BMC Psychiatry. 2024 Mar 27;24:233. doi: 10.1186/s12888-024-05657-8 (PMC10976762; doi:10.1186/s12888-024-05657-8)
Supplement: Supplementary file 1 — Supplementary Material 1 [file 12888_2024_5657_MOESM1_ESM.docx]

| **Supplementary Table 1. Correlation of the Birchwood Insight Scale items.** | | | | | | | | |
| --- | --- | --- | --- | --- | --- | --- | --- | --- |
|  | BIS 1 | BIS 2 | BIS 3 | BIS 4 | BIS 5 | BIS 6 | BIS 7 | BIS 8 |
| BIS 1 | 1 |  |  |  |  |  |  |  |
| BIS 2 | .11 | 1 |  |  |  |  |  |  |
| BIS 3 | .08 | .01 | 1 |  |  |  |  |  |
| BIS 4 | .11 | -.02 | .20* | 1 |  |  |  |  |
| BIS 5 | .05 | .04 | .51*** | .29*** | 1 |  |  |  |
| BIS 6 | -.03 | .14 | .20* | .27** | .13 | 1 |  |  |
| BIS 7 | .11 | .03 | .27** | .25** | .29*** | .32*** | 1 |  |
| BIS 8 | .08 | .12 | .20* | .39*** | .27** | .18* | .35*** | 1 |

**P* < .05; ** *P* < .01; ****P* < .001
